# Microfluidic Point-of-Care Devices: New Trends and Future Prospects for eHealth Diagnostics

**DOI:** 10.3390/s20071951

**Published:** 2020-03-31

**Authors:** Jorge Ricardo Mejía-Salazar, Kamilla Rodrigues Cruz, Elsa María Materón Vásques, Osvaldo Novais de Oliveira Jr.

**Affiliations:** 1 National Institute of Telecommunications (Inatel), 37540-000 Santa Rita do Sapucaí, MG, Brazil; kamillacruz@geb.inatel.br; 2 Sao Carlos Institute of Physics, University of Sao Paulo, P.O. Box 369, 13560-970 Sao Carlos, SP, Brazil; elsa_materon@yahoo.com (E.M.M.V.); chu@ifsc.usp.br (O.N.d.O.J.); 3 Chemistry Department, Federal University of São Carlos, CP 676, São Carlos 13565-905, São Paulo, Brazil

**Keywords:** biosensors, point-of-care, lab-on-a-chip, artificial intelligence, wearable devices, Internet-of-things, big data

## Abstract

Point-of-care (PoC) diagnostics is promising for early detection of a number of diseases, including cancer, diabetes, and cardiovascular diseases, in addition to serving for monitoring health conditions. To be efficient and cost-effective, portable PoC devices are made with microfluidic technologies, with which laboratory analysis can be made with small-volume samples. Recent years have witnessed considerable progress in this area with “epidermal electronics”, including miniaturized wearable diagnosis devices. These wearable devices allow for continuous real-time transmission of biological data to the Internet for further processing and transformation into clinical knowledge. Other approaches include bluetooth and WiFi technology for data transmission from portable (non-wearable) diagnosis devices to cellphones or computers, and then to the Internet for communication with centralized healthcare structures. There are, however, considerable challenges to be faced before PoC devices become routine in the clinical practice. For instance, the implementation of this technology requires integration of detection components with other fluid regulatory elements at the microscale, where fluid-flow properties become increasingly controlled by viscous forces rather than inertial forces. Another challenge is to develop new materials for environmentally friendly, cheap, and portable microfluidic devices. In this review paper, we first revisit the progress made in the last few years and discuss trends and strategies for the fabrication of microfluidic devices. Then, we discuss the challenges in lab-on-a-chip biosensing devices, including colorimetric sensors coupled to smartphones, plasmonic sensors, and electronic tongues. The latter ones use statistical and big data analysis for proper classification. The increasing use of big data and artificial intelligence methods is then commented upon in the context of wearable and handled biosensing platforms for the Internet of things and futuristic healthcare systems.

## 1. Introduction

Microfluidics is the name given to the technology for the fabrication of microminiaturized devices containing channels and chambers, with scale dimensions in the order of 1 mm or less, to control the flow behavior of small volumes of fluids [1]. At these levels, the fluid-flow is laminar, i.e., without chaotic turbulence, allowing the control of transport and mixing of molecules to enable separation and detection of analytes with high accuracy and sensitivity [2]. Several applications of microfluidics can be envisaged, then, in biotechnology, chemical synthesis, and analytical chemistry [3,4,5]. In particular, such precise manipulation of molecular interactions allows for miniaturization and rapid processing of samples, with experimental techniques in lab-on-a-chip analytical platforms [6,7]. These point-of-care (PoC) devices are applied to detect analytes of biological interest [8,9,10] and may provide rapid diagnostics even in remote regions (near a patient) with limited-resource or non-existing healthcare settings [11,12,13,14]. This can be made possible with synergistic integration of PoC devices with mobile communications, e.g. the fifth-generation wireless communications (5G), and the Internet of things (IoT) technologies [15,16,17,18]. With fast Internet connectivity with low-latency, high bandwidth, cloud-based storage, and real-time extensive computing capabilities for billions of connected devices, one may expect the next-generation healthcare programs to be more patient-friendly and cost-efficient. Indeed, integration of microfluidic PoC devices with mobile connectivity for cloud computing and big data analysis [19,20] may lead to remote monitoring and control of diseases [21,22,23], improving the doctor–patient communication. Research today is also devoted to surpass the challenges associated with rural, remote, and underserved communities, where there is demand for low-cost devices that require no refrigeration or laboratory infrastructure. Despite extensive research efforts, there are several drawbacks to address. They include the increasing demand for microfluidic chips which requires economic, easier, and faster fabrication techniques. Multiplexed detection, i.e., quantifying several analytes at the same time, is another requirement to be fulfilled. Signal amplification for detection of analytes at ultra-low concentrations is another important point, which is being mostly addressed through the use of nanoparticles to capture the analytes for improved signals [24,25,26]. A major challenge in microfluidic PoC devices is the detection and separation of chiral enantiomers, which exhibit the same physical and chemical properties in achiral environments. Many strategies have been proposed to overcome these limitations, e.g., with porous poly(vinylidene fluoride) (PVDF) membranes adsorbed with bovine serum albumin (BSA) sandwiched between two poly(dimethylsiloxane) (PDMS) slabs for chromatographic high-resolution chiral separation [27]. Other approaches include microchip electrophoresis [28] and temperature gradient focusing (TGF) [29].

In this review paper, we advocate the importance of low-cost, efficient microfluidic devices for point-of-care (PoC) diagnostics and the IoT, i.e., the connection of any physical object to the Internet. We highlight the merging of technologies with artificial intelligence and big data analysis, for which sensing has become essential. Portable devices exploiting microfluidics are crucial to meet the stringent demands of such ubiquitous sensing, which have prompted the development of novel fabrication methods. In addition to surveying recent developments, we discuss the challenges in mass production of microfluidic systems. It is worth mentioning the many review papers discussing microfluidic devices for various topics, e.g. fabrication techniques and their integration with other technologies for biosensing, PoC diagnosis, and drug discovery and delivery [11,12,13,14]. The physics behind fluid dynamics involved in microfluidics has also been discussed in some of these previous reviews. To avoid repetition, in this paper, we concentrate on the trends and advances during the last two years, exemplifying the merging of microfluidic PoC devices with IoT applications for future computer-assisted diagnosis, which is already being applied for monitoring chronic neurological disorders such as Parkinson’s disease (PD) [30,31].

## 2. Recent Trends in Microfluidic Device Fabrication

Conventional fabrication methods of microfluidic devices, such as lithography [7], soft-lithography [32,33,34], plasma treatment [35], and chemical vapor deposition [36], require cleanroom facilities and trained personnel. This makes them inefficient in terms of costs and fabrication speed to satisfy the increasing array of applications. Moreover, materials for microfluidics need to be optically transparent, mechanically strong, thermally stable, easily modifiable, and amenable for mass production [1]. There is not a single material capable of satisfying all these requirements simultaneously. Hence, in general, a material is selected according to the application of interest. Plastics are cheap, flexible, and easy to control, seemingly suitable for general purposes. However, their photolithography process is expensive and strong solvent materials cannot be used, which limits their applicability [37,38]. Silicon, on the other hand, exhibits good chemical and thermal compatibility, but it can be expensive, fragile, and opaque to visible and ultraviolet light, thus limiting its use in optical-based applications [38,39,40]. Although optical transparency, chemical inertness, rigidity, and high temperature resistance turn glass into a good material for microfluidics, it requires slow and high-cost deposition techniques [41], making it unsuitable for mass production. Some approaches, such as soda-lime glass [38], were proposed to address these limitations, but the need of rigorous chemical cleaning and thermal treatment for proper activation constitutes a major hurdle.

**Figure 1:**
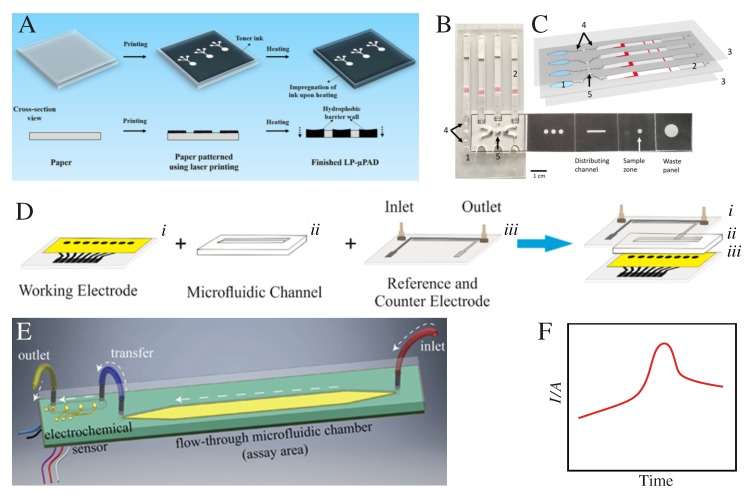
(**A**) Pictorial representation of a simple two-step procedure to fabricate LP-μPADs, consisting of printing and heating of the printed substrate. (**B**) An assembled paper-plastic microfluidic device for amplification and detection of malaria in blood. A paper origami device for DNA extraction and processing fabricated by hot wax printing method is attached. (**C**) Key features of the plastic microfluidic device: (1) manually pressed fluid chamber for fluid flow; (2) DNA detection strip; (3) safe handling (without evaporation) using acetate film covers; (4) filter paper-based valves for precise control of the LAMP reaction; and (5) filter paper for the LAMP reaction. (**D**) Schematics of the fabrication steps: (*i*) screen-printed carbon-based working electrodes; (*ii*) polystyrene card with double-sided adhesive with the microfluidic channel; and (*iii*) reference and counter electrodes with the inlet and outlet. (**E**) Illustration of the assay area and electrical connections of a unit cell of the fluidic arrangement in the printed circuit board electrochemical sensor. (**F**) Illustration of the amperometric detection mechanism for biosensors in (**D**,**E**). (**A**) was adapted with permission from Ref. [43]. Copyright 2019 Springer Nature. (**B**,**C**) are used with permission from Ref. [23]. Copyright 2019 National Academy of Sciences. (**D**) was adapted with permission from Ref. [46]. Copyright 2019 American Chemical Society. (**E**) was used with permission from Ref. [45]. Copyright 2018 MDPI.

Paper-based microfluidic devices appear to be advantageous for their cost effectiveness, capillary fluid-flow (power-free), high surface-area-to-volume ratio, and the ability to store reagents in active form within the fiber network [42]. Microfluidic channels have been produced through an automated laser printer deposition of hydrophobic ink on paper for PoC applications [43], as illustrated in Figure 1A. Permanent fixation of the hydrophobic barriers on the cellulose’s capillaries of the paper is reached upon heating at 165 °C for 15 min, as depicted in the cross-section view at the bottom of Figure 1A. These laser printed microfluidic paper-based analytical devices (LP-μPADs) were used to determine the nitrite content in aqueous solutions and artificial urine samples and for detecting *Escherichia coli* (ATCC 25922) through the Griess colorimetric assay. To detect biomarkers at ultra-low concentrations for early diagnosis in asymptomatic patients of diseases such as Parkinson’s disease, Alzheimer’s disease, cancer, and malaria, new strategies need to be combined with microfluidic PoC devices. One such strategy includes magnetic nanoparticles for signal amplification and capture of analytes [23,24], which also opens up the possibility for multiplex biomarker detection [25,26]. For example, Reboud et al. [23] combined magnetic nanoparticles, for lysis and analyte extraction, with a loop-mediated isothermal amplification (LAMP) [24] method for DNA diagnostics of malaria in rural communities. Difficulties associated with sample preparation and multiplexing were overcome through paper-folding origami techniques to distribute fluids both vertically and laterally. A picture of the assembled microfluidic paper-plastic PoC device is given in Figure 1B, which was used for fast and sensitive (more than current assays) detection of malaria. The paper strip attached on the right lateral side was used for DNA extraction and processing. Key features of the plastic microfluidic device, labeled 1–5, are shown in Figure 1C: (1) the buffer chamber activated by manually pressing [44]; (2) lateral flow DNA detection strip; (3) acetate films for safe handling and to avoid sample evaporation; (4) filter paper-based valves to avoid the LAMP reaction volume reaching the lateral flow strips after amplification; and (5) filter paper for the LAMP reaction. Although this approach has several advantages, e.g. versatility, high flow control, possibility of mass production, and suitability for different detection and quantification methods with small sample volumes, it has the main disadvantage of long optimization times.

Other proposals combine microfluidics, magnetic nanoparticles, and electrochemical analysis for precise, rapid, and highly sensitive detection of biomarkers [45,46,47]. Figure 1D depicts the fabrication steps of the microfluidic array using an inexpensive home cutter printer and low-cost materials for detection of breast cancer biomarkers [46]. The fabrication process comprises four main steps, labeled *i*–*iv* in Figure 1D. Another electrochemical device for amperometric enzyme-linked immunosorbent assay (ELISA) [45] had a microfluidic cell printed on a circuit board, as shown in Figure 1E. The assay area (microfluidic channel) consists of a polymer protected gold-strip connected to inlet and outlet ports. The device was used for cytokine detection through H${}_{2}$O${}_{2}$ depletion kinetics for diagnosing tuberculosis (TB). Figure 1F illustrates the amperometric results from the systems in Figure 1D,E. Although these microfluidic devices are cheap, portable, and environmentally friendly, further research is needed to integrate with microelectronic platforms for IoT-based PoC devices. The ever growing developers’ community of 3D printing platforms, extended library of printable materials, and low-cost printers is also making it possible to exploit 3D printing in freeing end-users from the know-how of manufacturers [48], especially with designs being available as open access data. Advances in 3D printed microfluidics are reviewed in Refs. [49,50].

## 3. Microfluidic Point-of-Care Devices

Reliable, rugged microfluidic systems for rapid and precise measurements on small sample volumes [10], without an expert operator [51], make it possible to develop chip-scale microfluidic PoC devices for highly precise laboratory operations. The various approaches and challenges for this aim using optical, electrochemical, and nanomaterials are reviewed in recent papers [6,52,53]. We will focus here on the most recent developments, in particular during the last two years.

**Figure 2:**
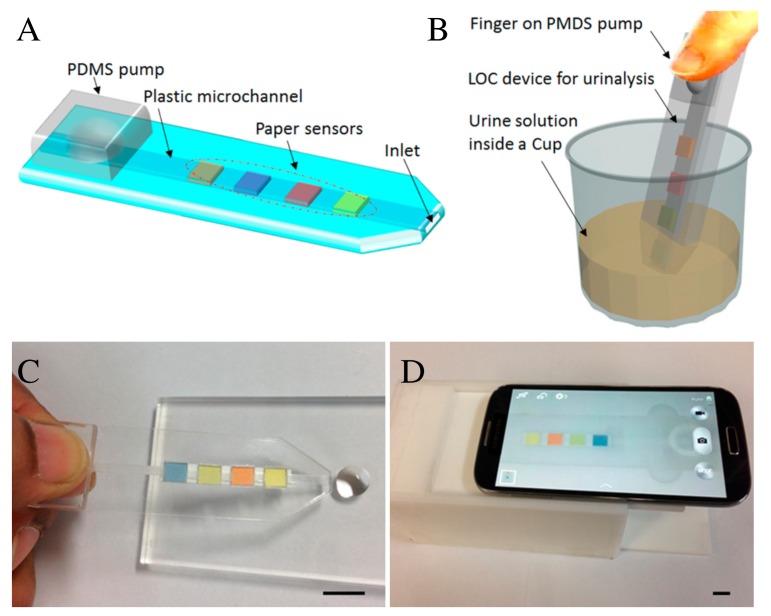
(**A**) Schematics of the hybrid point-of-care (PoC) microfluidic device. (**B**,**C**) The manual insertion (by finger pressing the pump) of the analyte into the reagent chambers from a sample container (a cup) or a drop. (**D**) A smartphone-based optical platform for colorimetric analysis. Reproduced with permission from Ref. [63]. Copyright 2017 American Chemical Society.

**Figure 3:**
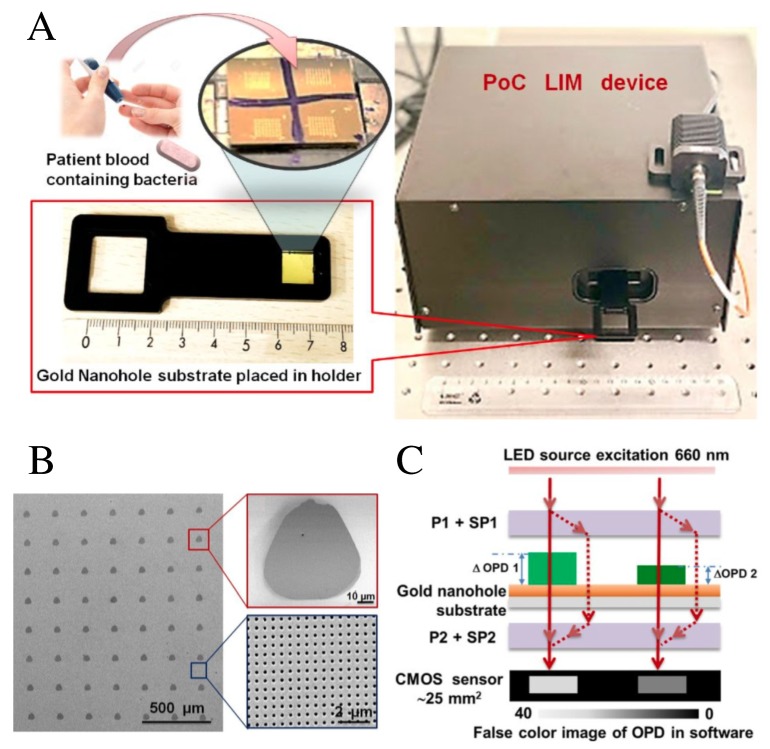
(**A**) Overview of the blood sampling and incubation onto the microarray biofunctionalized nanoplasmonic substrate for further analysis into the PoC LIM device. (**B**) SEM image of the 8×8 two-dimensional periodic bioprinted microarrays of protein G. The period length is 250 μm, as can be noted from the scale bar = 500 μm. The right upper-side and left lower-side insets show a zoomed view of a microspot with dimensions ≈ 50×55μm${}^{2}$ and the underlying gold nanohole array with 200 nm diameter and 600 nm for the period length. The plasmonic interferometric working principle is illustrated in (**C**). Reproduced with permission from Ref. [65]. Copyright 2019 American Chemical Society.

Paper-based colorimetric immunoassays are good candidates for low-cost diagnostic tools in resource-limited settings, but their application remains hampered by the need of desktop scanners that lack portability or of cameras that are sensitive to ambient light conditions. These limitations stimulated the use of smartphone-embedded high-resolution cameras to provide rapid optical detection of analytes with minimal instrumentation [54,55,56,57,58,59,60,61,62]. The time-dependent variation of the colorimetric assessment due to the lack of control in the reaction volume has been solved with a paper-plastic hybrid microfluidic device, integrating a single channel with a micropump [63]. The hybrid device is illustrated in Figure 2A,B. A finger-pressed PDMS (poly(dimethylsiloxane)) pump is used to fill the predefined volume reaction chamber with the sample (urine in this case), which reacts with an array of paper-based reagent test pads embedded across the plastic microchannel. An imaging box including PDMS light diffuser was used to distribute the light uniformly on the sample and improve device accuracy. The colorimetric change of the reagent pads upon reacting with the analytes is sensed by a smartphone camera, as shown by pictures in Figure 2C,D. Exotic optical properties of nanoscale-confined plasmonic fields are also being harnessed to develop portable microfluidic PoC devices [6]. In addition to CMOS (complementary metal-oxide-semiconductor) integrability of plasmonic nanostructures for chip-scale devices, this interest is fueled by the high sensitivity of surface plasmon resonances to small changes of the surrounding dielectric properties. This enables the label-free and real-time detection of chemical reactions near the metal-dielectric interface. The extraordinary transmission efficiency [64] of periodic sub-wavelength nanohole arrays in plasmonic films was used to produce a PoC device based on lens-free interferometric microscopy (LIM) [65] for label-free bacteria quantification, as shown in Figure 3A–C. Patient’s blood samples as small as 3 μL are required to perform a rapid one-step quantification of the analyte in this device (Figure 3A), where a LED (light emitting diode) source, overlapping the extraordinary transmission peak of the nanohole array in Figure 3B, is used to measure the optical phase shift. The latter measurement is performed as depicted in Figure 3C, where light travels across two polarizers P1 and P2, and two Savart plates SP1 and SP2.

**Figure 4:**
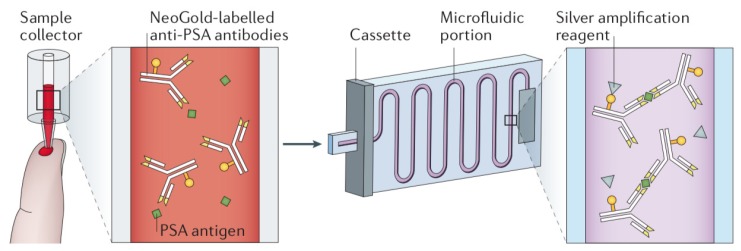
Overview of the microfluidic Sangia technology. From left to right: filling of the sample collector, which contains NeoGold-labeled anti-PSA monoclonal antibodies, and insertion of the sample into the microfluidic channel for silver amplification and detection.

The first microfluidic PoC device to determine the prostate specific antigen (PSA) for prostate cancer detection in less than 15 min was approved by the Food and Drug Administration (FDA) of the United States recently [66]. The device, named Sangia (Silver Amplified NeoGold ImmunoAssay) Total PSA Test (OPKO Health Inc.) [67], is illustrated in Figure 4. It consists of a microfluidic-based immunoassay assembled to a sample collector. NeoGold-labeled anti-PSA monoclonal antibodies are used in the sample collector and in the microfluidic portion to produce antibody–antigen–antibody sandwiches, which react with silver amplification reagents to yield a silver metallic film in the last step. The total PSA in the sample is measured through the optical density (in transmission) of the latter metallic film.

**Figure 5:**
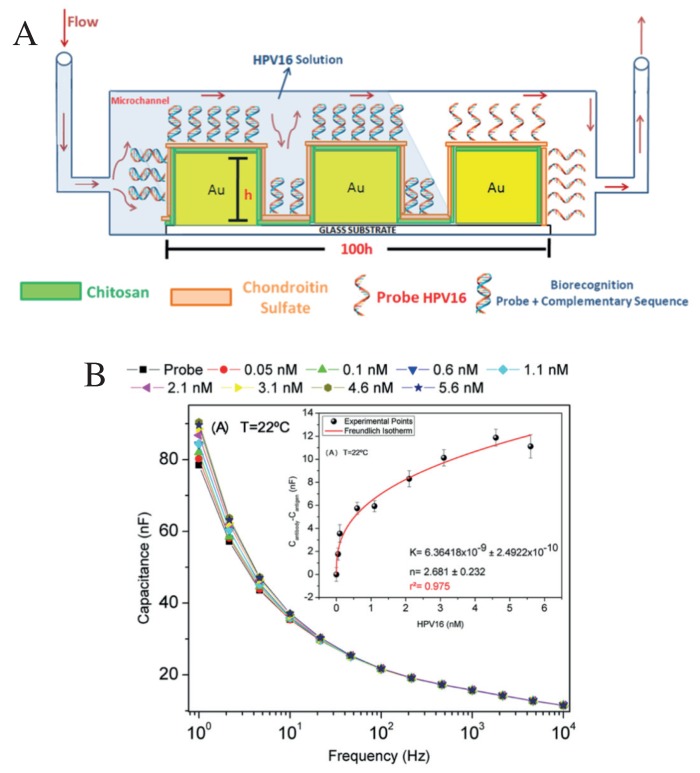
(**A**) Picture of the electrode functionalization and detection of HPV16 under continuous flow. (**B**) Capacitance spectra measurements at a hybridization temperature of 22 °C for different concentrations of ssHPV16-positive. The calibration curve is presented in the inset. Reproduced with permission from Ref. [68]. Copyright 2018 American Chemical Society.

**Figure 6:**
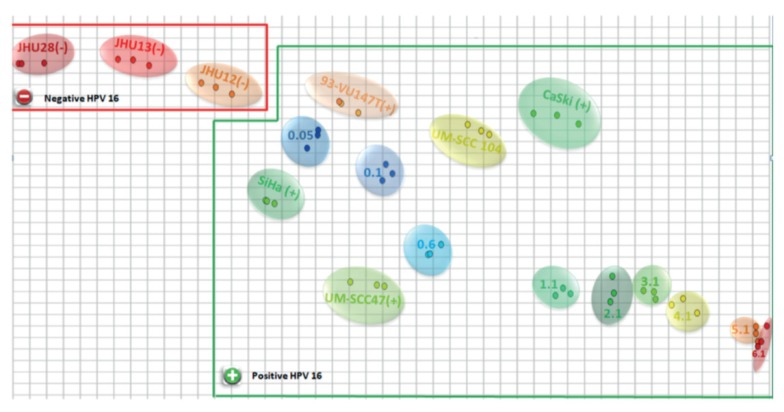
IDMAP plot of the capacitance vs. frequency measurements. Results projected inside the red line are for negative control, while the ones inside the green line are for positive results of HPV16. Reproduced with permission from Ref. [68]. Copyright 2018 American Chemical Society.

Figure 5 shows an example of a microfluidic device to detect the papillomavirus (HPV16) [68], an important etiologic factor in head and neck cancers. The device consisted of photolithographically manufactured gold interdigitated electrodes on glass BK7, represented in Figure 5A. The corresponding inlet and outlet holes are indicated. The gold electrodes were functionalized with layer-by-layer (LbL) films of chitosan (CHT) and chondroitin sulfate (CS). Figure 5B shows the capacitance curves for different concentrations of the complementary ssHPV16, measured at 22 °C, detected through the strong interaction with the probe chain (cpHPV16). The results in Figure 5B are only illustrative to show the working mechanism. Capacitance spectra of the cervical cancer cell lines CaSki and SiHa and the head and neck cancer cell lines JHU12, JHU13, JHU28, UM-SCC47, UM-SCC104, and 93-VU147T were analyzed using interactive document mapping (IDMAP), a nonlinear optimization technique that maps the data to a lower-dimensional space minimizing the error in the projection, to validate the manufactured genosensor, as seen in Figure 6.

Another approach for microfluidic PoC devices includes microfluidic electronic tongues (e-tongues), i.e., an electronic device mimicking the biological recognition of human tongue papillae for comparison of tastes. Such devices are made as arrays of sensing units whose multisensory units can be used to establish “fingerprints” from complex liquid samples [69], i.e., the recognition patterns in these devices can be used for identification/distinction of samples [70]. In contrast with the high specificity of other approaches, where pure or ultrapure samples are required, this concept could be more efficient for simultaneous multisensory devices. Proper classification is made merging statistical and computational tools to transform the large amount of data into different recognition patterns. Such an integration makes this approach promising for future eHealth systems with computer-aided diagnosis, where machine learning, big data, and IoT technologies should converge with biosensing capabilities. In particular, biosensing applications are being explored with e-tongues by functionalizing the building nanolayers with biomolecules to control the specificity in the interactions with the analyte under study [68,69,70,71,72,73,74,75,76]. This latter application can be used to collect huge amounts of patient’s biological data to feed machine learning algorithms, and then continuously convert them into knowledge (through big data and machine learning methods). With such an approach, one may achieve an efficient real-time online-based (IoT) discrimination of relevant threatening conditions and/or be able to adjust medications, which can be done through data collected from an Internet connected wearable biosensing device.

## 4. Microfluidic-Based eHealth

**Figure 7:**
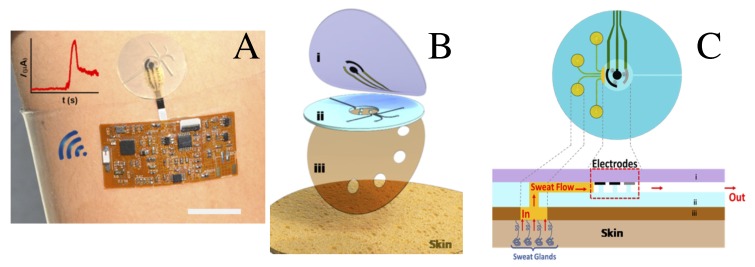
(**A**) Photograph of a wearable microfluidic device integrated with electronics for wireless communication. (**B**) Pictorial representation of microfluidic device composed of: (i) top PDMS layer with probing electrodes; (ii) PDMS microfluidic device; and (iii) adhesive layer on the skin. (**C**) Schematic representation of sweat collection and electrode operation on skin. Reproduced with permission from Ref. [88]. Copyright 2017 American Chemical Society.

The merge of information and communication technologies (ICTs) with PoC devices for continuous real-time health monitoring, for storage and processing of medical records (eHealth), has become a trend in biosensing technology [77,78,79,80,81,82,83,84,85,86,87,88,89,90]. Although it all started as a cost-effective alternative to deliver diagnostic strategies in the developing world, especially in rural, remote, and underserved communities, it has rapidly proliferated as a revolutionary tool that may enable virtual appointments between patients and doctors [17,18,19,20]. This has motivated the emerging field of epidermal electronics, i.e., the use of wearable simple stick-on tattoo that would function as a non-invasive sensing device for monitoring and communicating the vital physiological and metabolic functions of our body to a remote specialized center. An example of wearable epidermal electrochemical microfluidic device, integrated with a flexible electronic board enabling wireless real-time data transmission [88], is shown in Figure 7A. This trilayer microfluidic device consists of: (i) a PDMS layer with sensor electrodes; (ii) a PDMS microfluidic device with four channels and one outlet opening; and (iii) an adhesive layer (with four inlets) on the skin shown in Figure 7B. Capillary forces and glands pressure drive sweat flow through the microfluidic channel without external fields or pumps, as depicted in Figure 7C. An integrated bluetooth controller is used for continuous transmission (to a desktop application) of the amperometric detection of enzymatic reactions.

**Figure 8:**
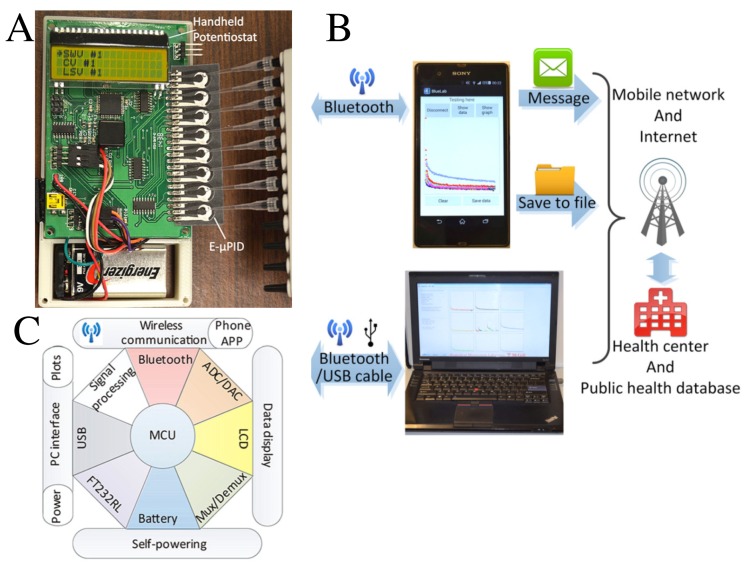
(**A**) Photograph and (**B**) architecture of a handheld potentiostat integrated with a paper-based microfluidic device for wireless communication of results to a smartphone or a computer application. (**C**) Illustrative representation of the communication of sensing results to the Internet or a health center for further processing. Adapted with permission from Ref. [85]. Copyright 2016 AIP Publishing.

Although μPADs are mostly based on optical detection principles, as noted from previous sections, they can be also used for electrochemical assays through electron transfer during redox reactions [91,92]. A device [85] with integrated WiFi and USB (Universal Serial Bus) capabilities for communication with computers and smartphones is shown in Figure 8A. A pictorial representation of the handheld potentiostat architecture is presented in Figure 8B. The electrochemical microfluidic paper-based immunosensor device (E-μPID) on the right-hand side of Figure 8A, functionalized for detection of immunodeficiency virus and hepatitis C virus antibodies in serum, exhibited high precision, sensitivity, and selectivity [85]. The transmission of detection results to remote sites or health databases, via Internet or mobile networks, as depicted in Figure 8C, can be used for telemedicine or patient’s health-data storage and analysis. This technology has been tested for geotagging and patient management of ebola virus survivors from the 2000–2001 Gulu outbreak in Uganda [90].

**Figure 9:**
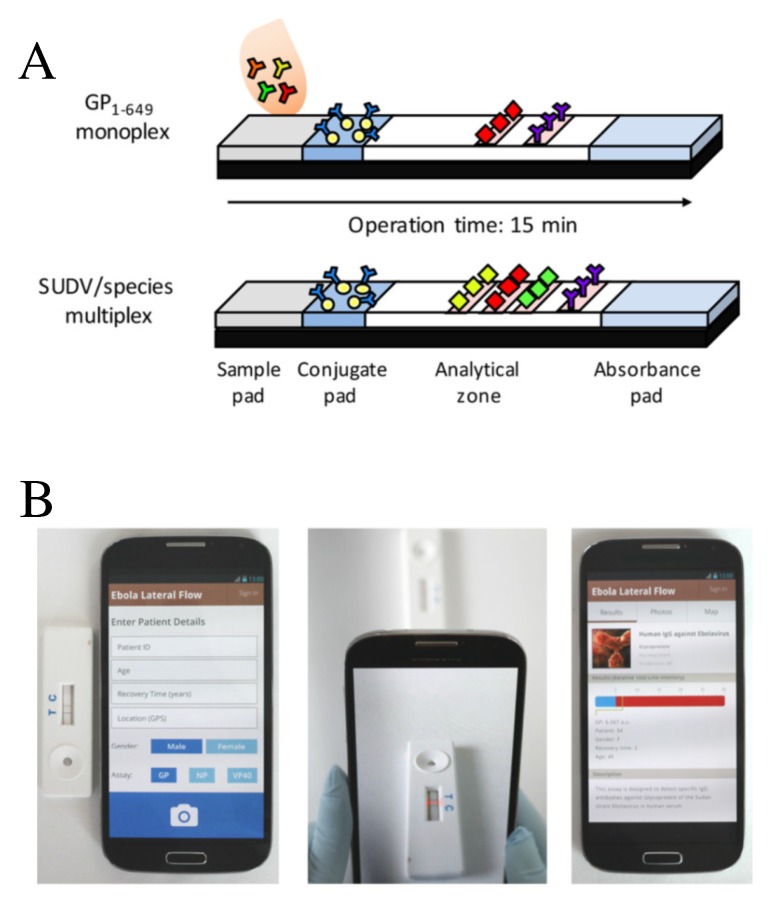
(**A**) schematics of monoplex and multiplex immunochromatographic strip configuration to detect Ebola virus. (**B**) Illustration of a smartphone application for a rapid, robust, simple, and portable device for the analysis of the strip results. A summary of the patient details can also be provided in this approach. Reproduced with permission from Brangel, P.; Sobarzo, A.; Parolo, C.; Miller, B.S.; Howes, P.D.; Gelkop, S.; Lutwama, J.J.; Dye, J.M.; McKendry, R.A.; Lobel, L.; Stevens, M.M. A Serological Point-of-Care Test for the Detection of IgG Antibodies against Ebola Virus in Human Survivors. ACS Nano **2018**, 12, 63-73 [90], which can be found at https://pubs.acs.org/doi/full/10.1021/acsnano.7b07021. Any requests for further permissions related to the material excerpted should be directed to the American Chemical Society.

**Figure 10:**
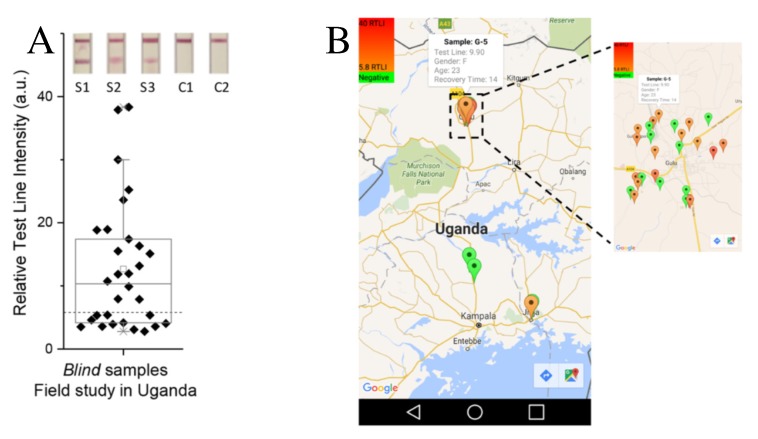
(**A**) Box plot of monoplex measurements of a field study in Uganda using GP${}_{1–649}$. The inset shows the microfluidic strips of three survivors (S1–S3) and two noninfected controls (C1–C2). (**B**) Map distribution for on-site testing of survivors of 2015 collection in Uganda. Reproduced with permission from Brangel, P.; Sobarzo, A.; Parolo, C.; Miller, B.S.; Howes, P.D.; Gelkop, S.; Lutwama, J.J.; Dye, J.M.; McKendry, R.A.; Lobel, L.; Stevens, M.M. A Serological Point-of-Care Test for the Detection of IgG Antibodies against Ebola Virus in Human Survivors. ACS Nano **2018**, 12, 63-73 [90], which can be found at https://pubs.acs.org/doi/full/10.1021/acsnano.7b07021. Any requests for further permissions related to the material excerpted should be directed to the American Chemical Society.

A test strip was designed for detecting EVD (ebola virus disease) IgG (immunoglobulin G) antibodies in the human serum. The first strip was a single analyte detection strip, referred to as monoplex GP${}_{1–649}$ in Figure 9A. The second one is a multiple-analyte detection, for which multiple recombinant proteins such as VP40 (matrix protein), NP (nucleoprotein), and GP${}_{1–649}$ are used (plotted as test lines). Improved sensitivity and specificity was reached using gold nanoparticles. The results are obtained through a smartphone application, which reads the colorimetric results from the strip and provides a positive or negative result based on a cutoff threshold. This biosensing approach has been the subject of several papers, where tools for registration, transfer, and analysis of assays data were discussed [93,94,95,96]. A summary of the patient details and a description of the test taken are given in Figure 9B. A box plot analysis of 30 blind serum samples from 25 survivors and 5 noninfected subjects (controls), obtained across Uganda, is shown in Figure 10A. These results were used as part of the assay validation in conjunction with other samples/measurements [90]. Insets in this figure correspond to readout results using GP${}_{1–649}$ monoplex strips for three survivors (S1–S3) and two noninfected controls (C1–C2). Figure 10B shows a geographical tagging of the samples together with the test result, represented by the color intensity of the red pins according to the semiquantitative response of each individual test, where red and green correspond to positive and negative results, respectively.

**Figure 11:**
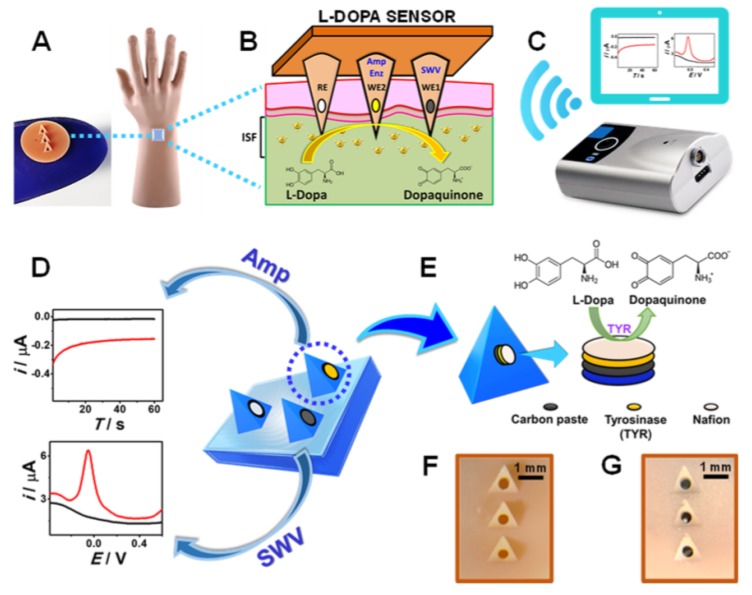
Schematics of the microneedle sensor array for L-Dopa detection: (**A**) microneedle sensor in a mannequin hand and (**B**) the L-Dopa sensor array. (**C**) Handheld wireless electroanalyzer for wireless data transmission. (**D**) Illustrative representation of the sensor array principle. (**E**) CP microneedle electrode, with the corresponding reagent layers: tyrosinase and Nafion. (**F**,**G**) Optical images of microonedles before and after assembling with CP. Reproduced with permission from Ref. [102]. Copyright 2019 American Chemical Society.

For Parkinson’s disease (PD), several approaches have been developed for wearable monitoring systems during the last years, with detection of bradykinesia and tremor [97,98,99,100,101]. Machine learning has been used to detect circadian rhythms and sleep, motor, and autonomic disruption, which is suitable for objective and non-invasive monitoring of PD patients [98]. These sensors do not use microfluidic principles, but the main concepts can be exploited with microfluidics. Indeed, a microneedle sensor has been employed for continuous monitoring of Levodopa in interstitial fluids [102], as illustrated in Figure 11. L-Dopa is an effective medication for PD treatment, which is metabolized to dopamine: the neurotransmitter lost in PD. Figure 11A shows the hollow microneedles with different carbon paste (CP) electrode transducers assembled into a two-working electrode microneedle array for parallel independent electrochemical probing of L-Dopa. Direct L-Dopa detection with square-wave voltammetry (SWV) using the microneedle WE1 is illustrated in Figure 11B, while the microneedle electrode (WE2) uses chronoamperometric measurements for tyrosinase (TYR)-based biocatalytic detection in Figure 11D. The reference electrode (RE) consists of an Ag wire, and no counter electrode was used. Data from these measurements are transmitted to a smart device or computer for further processing through a portable wireless electroanalyzer, as depicted in Figure 11C. Similar systems have been used for the continuous monitoring of glucose [103]. Figure 11E shows the amperometric detection of dopaquinone product after biocatalytic oxidation of L-Dopa at the tyrosinase enzyme embedded CP microneedle electrode. Optical images before and after assembling with the CP electrode are shown in Figure 11F,G. Other diseases such as diabetes are also targeted in eHealth diagnosis because of their relevance worldwide [103,104,105,106].

In this paper we present many approaches based on microfluidics, and we summarize their most relevant features in terms of analytes, detection method, and limits of detection in Table 1.

## 5. Summary, Outlook and Challenges

New categories of PoC devices, with significant progress in sensitivity and specificity, have been introduced during the last years. In particular, the integration with plasmonic capabilities and electrochemical measurements have been used for improved detection limits. Integration with ICTs for eHealth diagnosis has also been sought in biosensing, from wearable to handheld devices with WiFi enabled capabilities [107]. More specifically, the merging of complementary technologies, namely microfluidics surface functionalization, IoT, big data, and machine learning, allows one to envisage digital health advisors. They will have the ability to offer individualized diagnosis and therapy, at low cost in a fast, efficient way. In this context, a huge biomedical database for targeted treatment according to disease-specific symptoms and progression can be built. There are, however, multidisciplinary obstacles to overcome before optimized eHealth protocols can be reached: the first one is the integration of big data analysis for computer-aided diagnosis systems, where machine learning, data mining, and visualizations tools need to be further explored [17,18,19,20]. Security is also a key factor to be faced to avoid exposure of patient data, while providing better insights of the health status [108]. The second major challenge is the development of new materials and devices allowing reuse of microfluidic systems [109,110], to be cheaper and environmentally friendly.

The synergistic use of optics and microfluidics can enable new functionalities without loss of integrability or compactness [111]. In particular, optical forces for non-contact particle manipulation, sorting, and analysis [112,113,114,115,116,117] could provide a route to apply microfluidic drug delivery and screening [14] in chiral enantiopure pharmaceutics. This is desired to avoid toxicity, with improved excretion and potency [118]. Furthermore, it can also aid advances for cell separation, organ-on-a-chip, and cell culture for tumor-treating [119,120].

## Figures and Tables

**Table 1:** List of microfluidic devices reported in the literature to detect various analytes and diseases. Also given are the methods of detection and the limits of detection (LOD).

Detection Target	Method	μ-Fluidic Device	LOD
Nitrite and Escherichia Coli [43]	Imagen Capture	Paper-based microfluidic device	7 μM for Nitrite and 104 CFU/mL for E. Coli was
Malaria [23]	Naked eye	Microfluidic lateral flow	Presence-absence test
Breast cancer biomarker [46]	Electrochemical amperometric	Microfluidic immunoarray device	60 μU mL${}^{-1}$
Cytokine [45]	Amperometric	Microfluidic assay-cell on a printed circuit board	40 pg mL${}^{-1}$
Urine analysis [63]	Colorimetric/ smartphone	Paper-plastic hybrid microfluidic lab-on-chip	No data
E. coli [65]	Optical/plasmonic	Lens-free interferometric microscopy	8 cell mL${}^{-1}$
Papillomavirus [68]	Electrical/impedance spectroscopy	Microfluidic interdigitated electrodes	10.5–60.2 pM
Lactate/glucose [88]	Electrochemical amperometry	Microchip device. Photolithographic wearable microfluidic device	50 μM
Human immunodeficiency virus and hepatitis C [85]	Electrochemical	Paper-based microfluidic device	300–750 pgmL${}^{-1}$
Ebola [90]	Colorimetry	Lateral flow point of care test	Positive/negative
Levodopa/parkinson’s disease [102]	Chronoamperometry Square waves voltammetry	Microneedle sensor array	0.25–0.5 μM
